# Molecular Tooth Decay

**DOI:** 10.1371/journal.pgen.1000655

**Published:** 2009-09-18

**Authors:** Rob DeSalle

**Affiliations:** American Museum of Natural History, New York, New York, United States of America; Fred Hutchinson Cancer Research Center, United States of America

Charles Darwin was intrigued with vestigial organs and, in fact, made them a part of his “long argument” for the existence of evolution in the natural world in Chapter 13 of *On the Origin of Species*. Now on the 150^th^ anniversary of the publication of that seminal work, a study linking vestigial organs with molecular biology with paleontology has shown even more clearly how vestigial organs are of critical explanatory power in evolutionary theory. In the current issue of *PLoS Genetics*, Meredith et al. [1] present a comprehensive study of the Enamelin (*ENAM*) gene in placental mammals that sheds light on the evolutionary process. What is different about this study from others in the literature using gene sequences in the study of evolution is that *ENAM* sequences and functionality directly mirror the evolution of teeth in living and extinct placental mammals.

## Enamel, the Paleontologist's Friend

**Figure 1:**
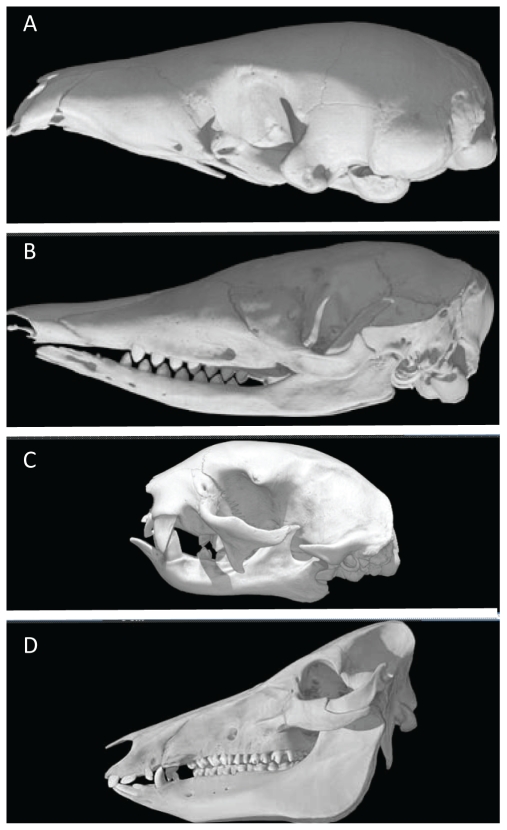
Skulls of toothed, toothed but enamelless, and toothless mammals. T = Toothed, T−E = Toothed without Enamel, and X = Toothless. (A) *Manis pentadactylus* (X, DigiMorph), (B) *Dasypus novemcinctus* (T−E, DigiMorph); (C) *Choloepus hoffmanni* (T−E, DigiMorph); (D) *Sus scrofa* (T, DigiMorph). The DigiMorph images are derived from http://digimorph.org/.

Enamel is one of several molecules that make paleontologists happy. It is the hardest substance found in vertebrates and is involved in tooth structure, as it forms the outer cap of teeth. It preserves extremely well in the fossil record and is oftentimes the only thing left in a preserved extinct mammal species. While the development of teeth is somewhat complex [2],[3],[4], the role of ENAM is well-known. It serves as an extracellular matrix protein that directs the formation of hydroxyapatite crystals during the formation of normal enamel. *ENAM* therefore becomes an important protein to follow in the transition of organisms with and without teeth, and teeth with and without enamel. What then becomes interesting is the distribution of enamel in living and extinct mammals (Figure 1). There are many toothless (edentulous) mammals and many mammals without enamel in their teeth. Ultimately, all of these mammals that depart from having enamel in their teeth or are toothless are derived from a common ancestor with enamel in its teeth. This latter fact allows for a detailed examination of the vestigial nature of *ENAM* in the mammals.

## Paleontology, the Molecular Biologist's Friend

**Figure 2:**
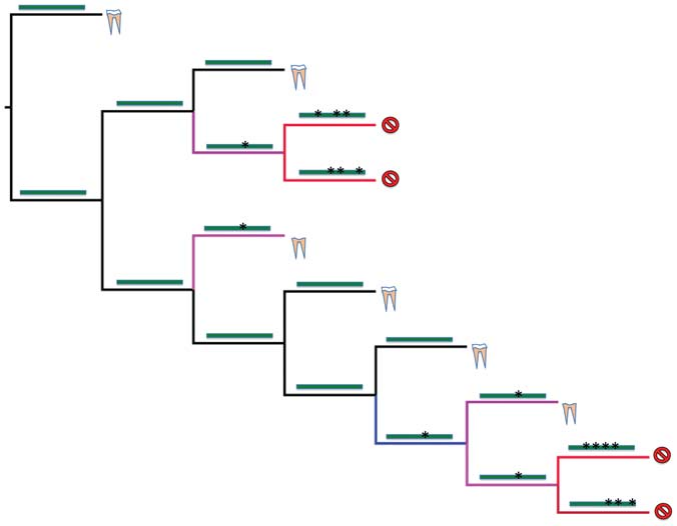
Hypothetical tree showing relationships of four toothed mammals (yellow and white components represent a full tooth), three enamelless tooth mammals (lack of white element represents loss of enamel), and four edentulous (toothless) mammals (slashed circle). Four branch types are then placed onto the tree using character reconstruction of the tooth anatomy and reconstruction of the *ENAM* gene. Functional branches (black) are those that have terminal nodes with a species with enamel or an internal node that was reconstructed as having had enamel. By definition, these branches will have genes on them that are reconstructed as having no stop codons or frameshifts. All other branches that are reconstructed as not having enamel in teeth can be premutation, mixed, or pseudogenes. In addition, any internal node that was reconstructed as not having enamel can be premutation, mixed, or pseudogenic. The presence or absence of stop codons or frameshifts in the *ENAM* gene was then used to establish the reconstructed state of these branches. In the figure, an uninterrupted gene has no asterisks on it while a gene with stop codons or frameshifts shows an asterisk. These branches are categorized as pre-mutation (blue) if the *ENAM* gene on the branch had no stop codons or frameshifts. A mixed branch (purple) contains the first detected occurrence of a frameshift mutation or stop codon in *ENAM*. And finally, a branch is a pseudogene branch (red) if it postdates the first occurrence of the frameshift or stop codon.

The authors used phylogenetic and selection detection approaches to comprehensively analyze the evolution of *ENAM* sequences in placental mammals. The first major result they discovered is that in all edentulous and enamelless mammals the *ENAM* genes are riddled with stop codons in all three reading frames and several frameshifts exist in the gene sequences. The second major result the authors uncovered required making two adjustments with how phylogenetic and selection detection analyses are done. First, they took their phylogeny and categorized branches in the tree into four categories—functional, pre-mutation, mixed (functional + pseudogene), and pseudogene—by reconstructing the presence and absence of enamel (Figure 2). Accomplishing these categorizations required a strong understanding of the anatomy of these animals from the fossil record and from reconstruction of genes on the branches of the phylogeny. Second, most studies using selection analysis as a tool usually look for increases in positive Darwinian selection (when dn/ds = ω>1.0) as an indicator of adaptive evolution (Box 1). In the present study, the lack of positive Darwinian selection, or what is called “purifying selection” (dn/ds = ω<1.0) relative to the neutral expectation (dn/ds = ω = 1.0), is used as an indicator of functionality.

**Box 1. A Simple Guide to dn/ds Values**

Selection studies at the molecular level count the number of replacements (changes at the nucleic acid level that also change the amino acid sequence after translation) and silent substitutions (changes at the nucleic acid level that do not alter an amino acid after translation) when comparing sequences. Using well-developed methods to correct these counts for molecular evolutionary processes, they then produce a ratio of the replacement site count to the silent site count called ω. This ratio can then be interpreted to indicate three kinds of selection pressure on molecules. If ω<1.0, then the molecule or sites being studied are under what is called “purifying selection”. When ω = 1.0, the sites are changing neutrally, and when ω>1.0 the sites are changing under what is called “positive Darwinian selection”. The first category simply retains amino acid sequence as a result of strong selection to stay the same. The latter category usually results in adaptation of the molecule to a changing external environment.

## Brush Your Branches—Molecular Tooth Decay

When the branches that are categorized as functional are examined, ω = 0.51 and is significantly less than the neutral expectation. Branches categorized as pseudogenes have an ω = 1.02, and branches categorized as premutation and mixed have intermediate values (0.83 and 0.98, respectively). These results indicate strong purifying selection on branches that lead to functional *ENAM* genes in the phylogeny and more and more relaxed purifying selection on the premutation and mixed lineages. Finally, when we reach pseudogene lineages, the pattern of evolution is neutral or totally relaxed and leads to what the authors suggest is “molecular decay”—the accumulation of stop codons and frameshift mutations. The molecular decay is then easily correlated with the loss of phenotype (i.e., loss of teeth and enamel).

## Dollo's Law and False Teeth

While the Meredith et al. [1] analysis presents compelling evidence for multiple cases of tooth and enamel loss and *ENAM* decay in the evolution of placental mammals, not a single instance of regain or re-evolution of enamel occurs once *ENAM* has decayed on an earlier branch of the tree. These observations are consistent with Dollo's Law that states that once a complex organ is lost it can never be regained in exactly the same form. Indeed, other workers [5] have used Dollo's Law to predict the range of time a pseudogene evolving neutrally could recover and “reactivate”. Their estimate was between 0.5 and 6.0 million years (MY) for genes of length up to 2 kb. While the estimates of potential recovery time using this approach have large variances, Meredith et al. [1] suggest that under the best assumptions the probability of *ENAM* recovering its function after 10 MY is only 0.014. The observed rapid demise of *ENAM* as demonstrated by the accumulation of stop codons and frameshifts in the genes of edentulous and enamelless mammals is entirely consistent with these calculations. As with decay and our own teeth, once an *ENAM* gene decays for a period of time, there is no resurrecting it. Any tooth that might evolve after a loss would not really be a tooth but rather an analogy of a tooth or a “false” tooth.

## Darwin's Vestiges As “Letters in a Word”

Darwin created a stunning metaphor for vestigial organs in Chapter 13 of *On the Origin of Species*: “Rudimentary organs may be compared with the letters in a word, still retained in the spelling, but become useless in the pronunciation, but which serve as a clue in seeking for its derivation” [6].

The metaphor suggests that rudimentary organs are like words spelled the same as their antecedents that have become pronounced differently, and with the different pronunciation the unpronounced letters in the spelling become useless in communication. Meredith et al. [1] amend this metaphor with their study. Vestigial organs are coded for by genes that are like words that have accumulated changed letters and misplaced spaces. The altered letters and misplaced spaces cause the words to decay and lose their meaning. In a similar manner, change in the “spelling and punctuation” in *ENAM* genes produces pseudogenes that are correlated with the loss or alteration of phenotype and the “decay” of teeth in certain lineages during mammalian evolution.
